# Inconsistency in the strength testing of dental resin-based composites among researchers

**DOI:** 10.12669/pjms.291.2922

**Published:** 2013

**Authors:** Naresh Kumar

**Affiliations:** Dr. Naresh Kumar, PhD, Assistant Professor, Science of Dental Materials Department, Institute of Dentistry, Liaquat University of Medical & Health Sciences, Jamshoro, Pakistan.

**Keywords:** Resin composite, Strength, Deformation rate, Mould variability, Weibull modulus

## Abstract

The aims of this paper were to review the current strength testing methods of the dental resin-based composites (RBCs) and to explore the inconsistencies with regard to strength testing among researchers.

***Data selection/extraction: ***An outline of the most relevant aspects of RBCs was created, and a subsequent literature search for articles published during last four decades (1970-2010) was conducted using the databases, namely PubMed, Science Direct and ISI Web of Knowledge.

***Conclusion: ***The literature review highlighted a lack of consensus among researchers regarding the reliability of ISO recommended three-point flexure strength testing method. Several investigators have used Weibull statistics for the analysis of RBCs strength data, however their applicability might be questioned as many RBCs contain greater resin content and may exhibit sufficient viscous deformation prior to brittle failure. In addition, variability in the selection of cross-head speed and mould material for strength testing was evident which may lead to variation in the strength data and render the interpretation difficult among researchers.

## Introduction

 The mechanical strength of dental resin-based composites (RBCs) is reliant upon the complex intra-oral forces such as compressive, tensile and shear introduced during mastication^1^ and it has a significant influence on the performance of dental restorations.^2^ The reproduction of such complex stresses in vitro is likely to be difficult in terms of cost and methodology. In addition, the dynamic tests may increase the probability of inertial effects and lead to misleading data. Consequently, various static-load-to-failure strength testing techniques i.e. compressive, diametral tensile, and flexure (bending) have been employed for the determination of the mechanical strength of RBCs.

 Now-a-days, flexure testing methods are being increasingly used for the evaluation of strength property of RBCs. However, applicability of each testing method is questioned in literature. Thus aims of this review paper were to highlight the pros and cons of each flexure testing method and to explore the variability in the strength testing of RBCs among investigators which may aid the selection and standardization of an appropriate strength testing method across the research community.

***Three-Point Flexure Testing:***

 The International Standards Organization (ISO) recommends the three-point flexure test to determine the flexural strength of RBCs (ISO 4049, 2000)^3^ which is frequently employed for dental RBC research worldwide. The three-point flexure test utilizes bar-shaped specimens (25 mm length, 2 mm width, and 2 mm thickness) and specimens are centrally loaded using a knife-edge indenter with a support span of 20 mm at the crosshead speed of 0.75±0.25 mm/min. The three-point flexure test produces tensile stresses on the lower convex surface of specimen. A disadvantage of the three-point bending test is that undesirable edge failures of specimen may occur, which may lead to an error in strength measurements.^2^ Also, due to the greater length of specimens compared with the exit window diameter of all handheld curing-light tips, an overlapping light-curing procedure is employed for the polymerization of specimens. This curing procedure may lead to an inhomogeneous curing as overlapped areas of specimens are likely to be polymerize greater than adjacent regions^4^^,^^5^ and decrease the reliability of flexure strength data.^5^^,^^6^ In order to eliminate inconsistent polymerization of bar specimens, various alternative methods have been suggested.

 Mehl et al^7^ and Manhart et al^8^ cured the bar-shaped specimens (25 mm length) with three light-units which were placed close to each other and operated simultaneously. Ferracane et al^9^ has suggested the use of oven-light curing units for irradiation of bar-shaped specimens, which may allow efficient and simultaneous polymerisation of multiple specimens. Yap and Teoh^4^ employed a shorter bar-shaped specimen (12 mm length) in order to achieve uniform curing in a single irradiation and authors have suggested a clinical relevance and easy fabrication of short bar-shaped specimens compared with long specimens.

 Despite the improved polymerization of specimens, edge fracture of specimen and resultant variation in strength values remain a disadvantage of the three-point flexure test. Moreover, the large specimen geometry is not representative of the restorations placed clinically.

***Four-Point Flexure Testing:***

 The four-point flexure test also employs similar bar-shaped specimens as the three-point flexure test. The specimens are loaded symmetrically at two locations with loading rollers and the distance between loading points is usually one-third or one-fourth of the support span length. In four-point flexure test, maximum bending occurs between the loading points, whereas in three-point flexure test, the maximum bending occurs below the loading roller. Hammant^10^ stated that four-point flexure test generates uniform stress field along the surface and reduces the stress concentration near the loading points. Moreover, the results of four-point flexure tests are likely to be more representative of the bulk properties since a greater portion of specimen is stressed. Despite these advantages, four-point flexure test has not been used frequently due to experimental difficulties, which may include the complex test fixture in contrast to three-point flexure test.^11^

***Bi-axial Flexure Testing:***

 Bi-axial flexure testing is a commonly used technique for the evaluation of dental ceramics.^12^^,^^13^ The main advantage of the bi-axial flexure test is that the maximum tensile stresses occur within the central loading area and spurious edge failures are eliminated in contrast to three-point flexure testing. The bi-axial flexure test has also been employed for the assessment of RBCs.^5^^,^^6^^,^^14^ A disc-shaped specimen (12mm diameter, 1 mm thickness) is usually used for bi-axial flexure test, which represents the average width of molar teeth and also allows a clinically relevant single-shot irradiation protocol instead of an overlapping cure used for bar specimens in three and four-point flexure testing. Furthermore, the results achieved by bi-axial flexure testing are also independent of specimen geometry and flaw direction.^2^

***Deformation rates for resin-based composite testing:***

 Deformation rates (cross-head speeds) during strength testing vary widely between studies (Table-I), which may lead to difficulty in comparison of results between operators. RBCs experience cyclic loading of varying magnitudes during their clinical life due to forces from mastication. Para-functional habits, such as bruxism, result in RBCs being subjected to constant forces for several minutes in contrast to the intermittent forces in normal mastication. Moreover, the effect of deformation rate on mechanical properties of polymer-based materials has been reported.^15^^,^^16^ However, mechanical properties of RBCs have been usually determined at one deformation rate and even ISO 4049 has suggested a limited range (0.75±0.25 mm/min) for the determination of flexural strength of RBCs. A reason for the selection of a lower deformation rate for mechanical testing of RBCs may be the occurrence of inertial effects at higher deformation rates. It is believed that inertial responses of the testing machine increase with increasing test speed, which may lead to erroneous results and difficulty in interpretation of data. Therefore, accurate characterization of machine compliance for deformation rate associated studies is important and should be conducted.

 It is clear that mechanical testing of RBCs at one deformation rate may not provide sufficient information to elucidate the material behaviour in the real clinical environment. Thus determination of mechanical properties of RBCs with regard to deformation rate should be standardized.

**Table-I:** Cross-head speeds used in some mechanical tests for resin-based materials studies

*Reference*	*Year*	*Test type*	*Cross-head speed (mm/min)*
Aguiar et al.^27^	2005	Diametral	10.0
Beun et al.^28^	2007	Three-point flexure	0.75
Chabrier et al.^29^	1999	Compression	0.2
Curtis et al.^14^	2008	Bi-axial flexure	1.0
Deepa and Krishnan^30^	2000	Compression Diametral	10.0
Ferracane et al.^31^	1998	Fracture toughness Three-point flexure	0.13 0.254
Kim et al.^32^	1994	Three-point flexure	0.1
Labella et al.^33^	1994	Three-point flexure	5.0
Lohbauer et al.^34^	2003	Four-point flexure	0.75
Manhart et al.^10^	2001	Three-point flexure	0.5
Peutzfeldt and Asmussen^17^	2000	Diametral Three-point flexure	10.0 1.0
Pilliar et al.^35^	1987	Fracture toughness	5.0
Sabbagh et al.^36^	2002	Three-point flexure	0.75
Sandner et al.^37^	1997	Three-point flexure	5.0
Tian et al.^38^	2008	Three-point flexure	0.5
Yesilyurt et al.^39^	2009	Three-point flexure	0.05

***Mould Variability:***

 The International Standard Organization specifies the stainless steel moulds for the fabrication of bar-shaped specimens (ISO 4049, 2000).^3^ However, different mould materials, stainless steel,^4^ namely black nylotron,^5^ brass,^17^ aluminium,^18^ and teflon^19^ have been utilized for specimen preparation prior to flexure strength testing. The mould material type may affect the depth of cure of RBCs specimens and resultant flexural strength data.

 A high proportion of light can be transmitted through the structure of translucent white polytetrafluoroethylene (PTFE). On contrary, metallic and black nylotron moulds block the light transmission through the structure of material. In addition, the cavity walls of PTFE and metallic moulds can reflect the light. Harrington and Wilson^20^ compared the influence of three mould materials, namely PTFE, stainless steel and black nylotron on the depth of cure of commercial RBC specimens. RBC in the PTFE mould exhibited a greater depth of cure compared with the black nylotron and stainless steel moulds whereas no significant difference between the depth of cure of the black nylotron and stainless steel was identified. The results of PTFE moulds were likely due to a significant amount of light transmission through the structure of mould. Hence, in order to achieve meaningful comparison of data, standardization of the mould material is required.

***Weibull Modulus:***

 Fracture of brittle materials usually originates from flaws distributed at the surface or within the material. The major flaw size, on which the strength of a material is based, varies from specimen to specimen and therefore a variation in strength values is expected. However, the strength data of RBCs has been mainly reported by only mean strength values and associated standard deviations and it is assumed that mean strength is a true value and signifies a normal strength distribution. In reality, the defect population lacks this level of homogeneity and as a result the failure of material may occur at lower stresses.^21^ Therefore, the strength of RBCs may only become meaningful when it is evaluated by a probability function such as Weibull statistics.^21^

**Table-II:** Weibull modulus (m) of different RBCs identified in some studies

*Reference*	*Year*	*Test method*	*Materials*	*Weibull modulus* *(m)*
Curtis et al.^24^	2009	Bi-axial flexure	Heliomolar Filtek Z250 Z100 Filtek Supreme Body Filtek Supreme Translucent Grandio Grandio Flow	5.1-8.2 4.3-11.5 3.3-10.8 4.0-11.8 6.0-16.9 7.3-12.1 2.1-9.5
Ilie and Hickel^40^	2009	Three-point flexure	FiltekSilorane EsthetX Tetric Tetric Ceram Tetric Ceram HB TetricEvoCeram Filtek Supreme XT	9.0-11.4 6.5-10.1 8.5-10.1 5.3-14.9 3.5-15.4 13.5-17.8 3.6-9.4
Lahbauer et al.^34^	2003	Four-point flexure	Charisma Definite Filtek Z250 Heliomolar Solitaire Solitaire II Surefil Tetric Ceram	9.2 9.1 10.8 8.1 5.6 9.6 8.4 12.3
Palin et al.^5^	2003	Bi-axial flexure	Oxirane-based RBC Filtek Z250 Z100	16.2 11.9 10.2
Palin et al.^5^	2003	Three-point flexure	Oxirane-based RBC Filtek Z250 Z100	9.2 8.5 6.3
Palin et al.^6^	2005	Three-point flexure	Z250	8.5-10.1
Palin et al.^6^	2005	Bi-axial flexure	Z250	11.9-12.4
Pick et al.^25^	2010	Three-point flexure	Concept Advanced Filtek Z250 Heliomolar	3.9 4.2 3.3
Pick et al.^25^	2010	Bi-axial flexure	Concept Advanced Filtek Z250 Heliomolar	8.6 6.6 7.2
Rodrigues Junior et al.^23^	2008	Three-point flexure	Filtek Z250 Filtek Supreme	7.6 9.7

 The Weibull modulus of a group of specimens may consider the flaw population in a brittle material. A high Weibull modulus suggests a narrow distribution of defects and an increased reliability of strength data. Other useful features of Weibull statistics include its ability to predict changes in distributions according to the physical size of individual test specimen. By this property of Weibull statistics, strength values of one sample may be scaled to predict the corresponding strengths values for different sample size, shape or stress distribution.^22^

 Weibull statistics have been employed for strength data of RBCs in many studies.^5^^,^^6^^,^^18^^,^^23^^-^^25^ Palin et al^5^compared the reliability of bi-axial flexure test of RBCs with three-point flexure test using Weibull modulus and have suggested that bi-axial flexure testing method provides a more reliable testing method than three-point flexure. The increased reliability of bi-axial flexure testing was attributed to decreased curing variability in disc shaped specimens in contrast to three-point flexure specimens. Rodrigues Junior et al^23^ compared the four-point flexure strength of a nanofilled and a microhybrid RBCs by Weibull modulus and no significant differences between flexural strength and associated Weibull modulus of both RBCs were observed. The authors suggested that similar behaviour of RBCs might be a consequence of comparable filler content and morphology of both RBCs. Chadwick et al^26^ investigated the influence of placement technique on compressive strength of RBC using Weibull statistics. In one group RBC specimens were prepared with an amalgam plugger, while in the other group specimens were prepared by smearing with a plastic spatula. The specimens group prepared by condensation technique showed lower Weibull modulus, which is indicative of decreased reliability compared with specimens prepared by smearing technique.

 It is clear that there is a considerable interest in using Weibull statistics for the evaluation of RBC strength reliability. However, a wide range of RBCs with variable elastic moduli are available. Despite this fact, no one has considered the applicability of Weibull statistics with less brittle RBCs. Since Weibull statistics are well-established for highly brittle materials, it might be that RBCs with greater resin content may not provide strength data that is applicable to the use of Weibull statistics. Moreover, many studies have submitted RBC strength data to Weibull statistics and found a wide variation in Weibull moduli of similar RBCs. For example, a Weibull modulus of Filtek Z250 ranging between 4.2-12.4 has been reported in the literature (Table-II). These differences in results may lead to incorrect interpretation of data between investigators. Therefore, research in terms of applicability of Weibull statistics to different RBCs is required, which may consequently aid the accurate interpretation of data.

## Conclusions

 A lack of consensus about the strength testing method is evident amongst investigators. Thus, standardization of a single strength testing method is warranted so as to achieve meaningful comparison of data.

 The selection of deformation rate and mould material vary widely in literature, hence variation in the results is expected. There is a clear need for a general agreement on the standardization of strength testing with regard to selection of deformation rate and mould material.

 The use of Weibull statistics might be questioned for less brittle and viscoelastic materials such as RBCs compared with ceramic-based materials, for which Weibull statistics are established. Until now, no researcher has considered the effect of such characteristics on the applicability of Weibull statistics in RBC related research. Therefore, an investigation concerning the applicability of Weibull statistics in different classes of RBCs is required.
